# Comparison of Percutaneous Intramedullary Pin Fixation and Elastic Stable Intramedullary Nails for Pediatric Forearm Fractures

**DOI:** 10.7759/cureus.84180

**Published:** 2025-05-15

**Authors:** Alyssa Basdavanos, Matthew B Holloway, Trinity A Kronk, Amber McDermott, Richard P Steiner, Todd F Ritzman, Lorena V Floccari

**Affiliations:** 1 Department of Orthopedic Surgery, Harbor - UCLA (University of California, Los Angeles) Medical Center, Torrance, USA; 2 Department of Orthopedic Surgery, Summa Health, Akron, USA; 3 Department of Biomedical Sciences, Northeast Ohio Medical University, Rootstown, USA; 4 Department of Orthopedic Surgery, Aultman Hospital, Canton, USA; 5 Department of Orthopedic Surgery, Akron Children's Hospital, Akron, USA

**Keywords:** elastic stable intramedullary nails, pediatric forearm fractures, pediatric orthopaedic surgery, pediatric orthopaedic trauma, percutaneous pinning

## Abstract

Introduction: Percutaneously placed intramedullary pin fixation poses potential benefits to the patient in the treatment of pediatric forearm fractures; however, physicians still have concerns regarding clinical outcomes in comparison to traditional surgical management. The purpose of this study is to analyze the operative time, clinical outcomes, and costs associated with percutaneous intramedullary pinning (PIP) compared with elastic stable intramedullary nails (ESINs) in pediatric patients undergoing surgery for forearm fractures.

Materials and methods: In this single-center, retrospective comparative study, patients ≤18 years old with forearm fractures requiring surgical intervention with either PIP or ESINs were included. Operative times, treatment outcomes, complications, unplanned reoperations, and cost data were analyzed. A prospective phone survey was also conducted for patient satisfaction with PIP removal in the outpatient setting.

Results: A total of 140 patients were included in this study (26 PIP, 114 ESIN). PIP patients were significantly younger than ESIN patients (6.2 vs 11.9 years, p<0.001). After accounting for single- versus both-bone fixation, PIP operative time was 40 minutes shorter than that for ESIN (p<0.001). There was no statistically significant difference between the groups in fracture healing, complications, range of motion, or unplanned reoperations. Of the 38% (n=10) of PIP patients who responded to the phone survey, 100% preferred outpatient removal. Implant charges were 10.5 times higher for ESIN patients compared to PIP ($598 vs $52). There were no charges for outpatient pin removal in the postoperative global period, whereas elective ESIN removal in the OR had a mean charge of $10,836 (±3,891).

Conclusions: Our study supports the use of percutaneous pinning as a cost-efficient alternative to ESIN for diaphyseal forearm fractures in young pediatric patients with a mean age of six years. This technique avoids the need for surgical implant removal, decreases operative time, and significantly reduces costs without increasing complications or reoperations.

## Introduction

Diaphyseal forearm fractures of the radius and/or ulna are common pediatric injuries managed according to fracture morphology, alignment, and skeletal maturity. While most pediatric patients are successfully treated non-surgically with casting, some may require surgery, such as for an open fracture and/or malalignment that exceeds acceptable limits using age-based alignment criteria after attempted closed reduction. Patients with a surgical indication typically undergo either flexible intramedullary nail fixation or open reduction internal fixation (ORIF), depending on age and fracture morphology [1,2].

Percutaneously placed intramedullary pin fixation for surgical treatment of forearm fractures is used less commonly, but has been described using either Kirschner wires (K-wires) and/or Steinmann pins within the intramedullary canal [3-7]. The potential advantage of percutaneous pinning is that pins may be left exposed outside the skin and removed in the office, rather than surgically removed in the operating room, as is commonly performed for buried flexible intramedullary nails. Critics of percutaneous intramedullary pin fixation for forearm fractures believe that adequate diaphyseal fracture healing does not occur before the pin is removed at 4-6 weeks, thus leaving the patient prone to refracture and displacement [4,5].

Prior studies have demonstrated that percutaneous intramedullary pinning (PIP) of radial and/or ulnar diaphyseal fractures is safe and effective for the treatment of diaphyseal forearm fractures in children [4-10]. However, there are few studies directly comparing the differences in outcomes and cost between PIP versus buried elastic stable intramedullary nails (ESINs). The purpose of this study is to analyze the operative time, clinical outcomes, and costs associated with PIP compared with ESINs in pediatric patients undergoing surgery for forearm fractures.

## Materials and methods

This is a single-institution retrospective comparative study of consecutive patients treated for forearm fractures from 2012 to 2020. All patients were treated at a large, multi-campus, Level 1 trauma, free-standing children’s hospital. Patients ≤18 years old with surgical treatment of a diaphyseal forearm fracture utilizing an intramedullary implant, either ESIN or PIP, were included. This consisted of radial and/or ulnar diaphyseal fractures and/or Monteggia fracture dislocations. Surgical indication was at the discretion of the surgeon, typically for open fracture and/or failure to maintain acceptable alignment with a closed reduction attempt. Exclusion criteria were incomplete radiographic assessment, incomplete clinical and radiographic follow-up to fracture healing, and any ipsilateral upper-extremity injuries. 

After Institutional Review Board (IRB) approval of this study, the electronic medical records and radiographs were reviewed, and clinical and radiographic outcomes were analyzed. Additionally, a prospective phone survey, adapted from the Short Assessment of Patient Satisfaction measure, with verified reliability and validity [11], was conducted for patients who had PIP fixation. PIP patients ≥18 years old and/or parents/guardians of patients <18 years old were called to participate in a survey regarding their patient experience, satisfaction with surgical management, and pain scores during outpatient pin removal. If patients declined participation in the phone survey and/or were unable to be reached after weekly calls on three occasions, then they were excluded from the survey portion of this study.

Technique: PIP

**Figure 1:**
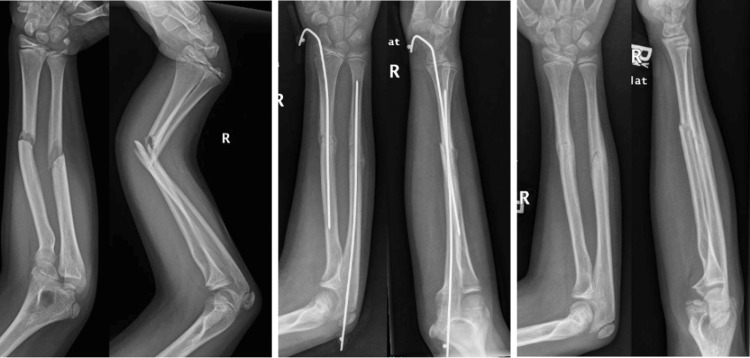
Preoperative, four-week postoperative and 10-week postoperative radiographs of an 11-year-old male treated with percutaneous intramedullary pinning of a right type 1 open both bone fracture

Percutaneous intramedullary pins were placed using a technique that has been previously described [3,10,12]. Radial shaft fractures are treated with a percutaneous starting point at the tip of the radial styloid with a transphyseal trajectory into the diaphysis. Ulnar placement is technically simple with direct access to the medullary canal via a percutaneous starting point in the olecranon tip. Steinmann pins utilized were either 0.062 or 0.078, dependent on intramedullary canal size and patient age. Pins are 9 inches (23 cm) long; thus, only patients with radius or ulna shaft fractures in which the Steinmann pins would adequately traverse the fracture were considered for PIP. Pins are covered with sterile dressing and cast padding under a long arm cast and removed in the office at four weeks postop. Following pin removal, patients are typically placed back into fiberglass casts for an additional two weeks for continued fracture healing (Figure 1).

Technique: ESIN

Patients treated with ESINs underwent a traditional, previously described technique [13]. For radial shaft fractures, a physeal-sparing entry point is utilized at either the radial styloid or dorsally through Lister’s tubercle. For ulnar shaft fractures, an anconeus starting point or proximal olecranon apophysis starting point is utilized. An entry site in the cortex is made with either an awl or a drill bit. The titanium nail is then carefully contoured to the radial bow or left straight for the ulnar shaft, and subsequently advanced into the intramedullary canal with the use of a T-handle device. Following closed versus open reduction of the fracture, the nail is advanced past the fracture site, and the tip is cut short to be buried subcutaneously.

Statistical methods

ESIN and PIP groups were compared for the entire cohort, and a separate subanalysis was performed on skeletally immature patients who were ≤10 years old. For categorical variables, the chi-square test of homogeneity and Fisher's exact test were used. For quantitative variables, the Wilcoxon rank sum test and the median test were used. Logistic regression models were used to test for associations between age group and categorical variables, conditional on treatment group. Quantitative variables were analyzed using nonparametric two-way analysis of variance based on aligned ranks. Statistical significance was defined as p ≤0.05.

## Results

This study included 140 patients (26 PIP, 114 ESIN) (Table 1). The PIP group was significantly younger than the ESIN group (6.2 vs 11.9 years old, p<0.001). The probability that a surgeon selects ESIN increases as the age of the patient increases (U=0.295, p<0.001). The odds ratio (OR) of ESIN = 1.6 (95% CI: 1.3, 1.9), indicating that the odds of using a buried nail increase by 60% for each additional year of age. There were no statistically significant differences between PIP versus ESIN groups in sex or incidence of open fracture (34.6% vs 30.7% open, p=0.698). Nine of the 26 PIP cases required an open reduction. 

**Table 1:** Differences in variables between the PIP and ESIN groups PIP, percutaneous intramedullary pinning; ESIN, elastic stable intramedullary nail.

Variable		PIP (n=26)	ESIN (n=114)	p-Value
		Median ± IQR (Range) or n (%)		
Sex (% female)		11 (42.3%)	34 (29.8%)	0.219
Age (years)		6.2 ± 4.3 (2.5-13.8)	11.9 ± 3.0 (0.7-18.5)	<0.001
Type of fracture (#, %)	Both bone forearm	9 (34.6%)	101 (88.6%)	<0.001
	Monteggia	17 (65.4%)	8 (7.0%)	
	Isolated radius	0	5 (4.4%)	
Type of implant (#, %)	Both-bone	10 (38%)	70 (61%)	0.013
	Ulna-only	16 (62%)	26 (23%)	
	Radius-only	0	18 (16%)	
Open fracture (#, %)		9 (34.6%)	35 (30.7%)	0.698
Duration of surgery (minutes)		39 ± 34 (14-85)	90 ± 50 (20-196)	<0.001
Time in cast (days)		44 ± 5.5 (21-65)	47 ± 26.5 (11-85)	0.015
Length of follow-up (months)		2.6 ± 1.4 (1.4-13.9)	7.7 ± 4.8 (1.1-63.1)	<0.001
Complications (#, %)		3 (11.5%)	10 (8.8%)	0.709
Unplanned reoperation (#, %)		1 (3.8%)	6 (5.3%)	>0.999
Surgical implant removal (#, %)		0 (0%)	88 (77%)	>0.999

The type of implant differed between the groups (p<0.001). There was a greater incidence of single-bone implant fixation in the PIP group (62% vs 39%) (Table 1). All patients with single-bone fixation in the PIP group (62%) had ulnar fixation of a Monteggia fracture, while single-bone fixation with ESIN included 16% radial shaft fixation and 23% ulnar shaft fixation of a Monteggia fracture. In the total population, patients undergoing single-bone fixation were significantly younger than patients with both-bone fixation (9.8 vs 12.7 years, p=0.004). 

**Figure 2:**
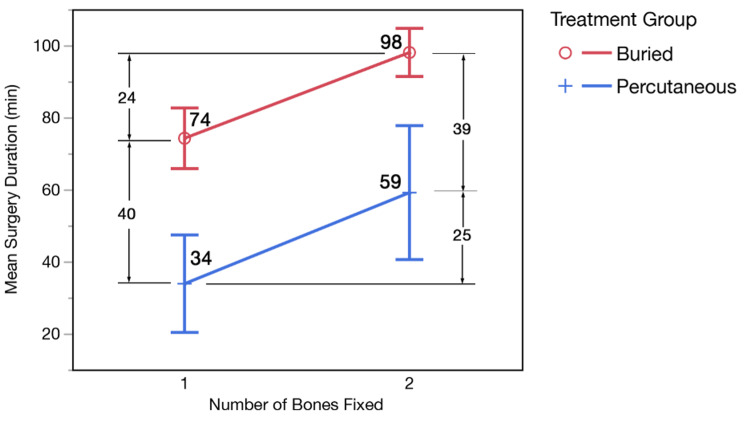
Mean surgery duration illustrating differences in the operative time between the percutaneous intramedullary pinning and elastic stable intramedullary nail groups, and between single-bone and both-bone fixation

Operative time was significantly shorter for PIP than that for ESIN (39 vs 90 minutes, p<0.001, Table 1). After accounting for single- versus both-bone fixation, operative time remained 40 minutes shorter in the PIP patients compared to that in the ESIN patients (p<0.001). The addition of fixation in the second bone lengthens the operative duration by about 25 minutes over single-bone fixation in both groups (p<0.001) (Figure 2). 

There was a significantly shorter time in cast for the PIP group (44 vs 47 days, p=0.015, Table 1). There were no significant differences in overall complication rates between PIP and ESIN groups (11.5% vs 8.8%, p=0.709), or in unplanned reoperation rates (3.8% vs 5.3%, p>0.999, Tables 1, 2). There was one reoperation in the PIP group for ulnar hypertrophic nonunion versus six reoperations in the ESIN group for three refractures, one ulnar nonunion, one deep infection, and one compartment syndrome after multiple attempts to cross the fracture site with the nail (Table 2).

**Table 2:** Complications and unplanned reoperations in the PIP and ESIN groups, excluding planned implant removals PIP, percutaneous intramedullary pinning; ESIN, elastic stable intramedullary nail; ORIF, open reduction internal fixation.

Group	Category	Complication	Age (years)	Treatment	Timing
ESIN (n=114)	Major (required reoperation)	Refracture	14.6	Revision ESIN	1 year after implant removal
		Refracture around nails	10.6	Revision ORIF, with subsequent refracture at distal end of plate	10 months
		Refracture around nails	14.9	Revision ORIF	3 months
		Ulnar nonunion	12.72	Revision ORIF	6 months
		Compartment syndrome	11.47	Compartment releases	1 day
		Deep infection at open fracture site	11.79	Irrigation and debridement	10 days
	Minor	Delayed union	12.68	Resolved with bone stimulator	7.5 months
		Olecranon bursitis	13.34	Conservative care, early implant removal	2 months
		Olecranon bursitis	13.90	Conservative care, early implant removal	2.5 months
		Implant prominence	10.83	Conservative care, early implant removal	2 months
PIP (n=26)	Major	Ulnar hypertrophic nonunion	6.86	Revision ORIF	3 months
	Minor	Olecranon bursitis	13.75	Conservative care, resolved	13 months
		Granuloma at pin site	2.49	Conservative care - silver nitrate applied, resolved	4 weeks

In a subanalysis of skeletally immature patients ≤10 years old, similar patterns were identified. PIP patients were still younger (6.1 vs 9.2 years, p<0.001) and had shorter operative time (29 min vs 81 min, p<0.001), though without difference in incidence of single bone fixation (PIP 81% vs ESIN 61%, p=0.109). There was also no significant difference in gender, incidence of open fracture, time in cast, or rate of complication and/or unplanned reoperation (all p>0.1). The ESIN group (n=33) had one refracture in patients ≤10 years old, while the PIP group (n=21) had no reported refractures.

When performing a subanalysis of patients with Monteggia versus non-Monteggia fractures between groups, patients with Monteggia fractures did not differ significantly in age, surgery duration, or length of follow-up. For patients with non-Monteggia fractures, there was no difference in age between the treatment groups; however, surgery duration was nearly 40 minutes longer for the ESIN group (90 min vs 53 min, p=0.002). Length of follow-up was also longer for the ESIN group compared to the PIP group (7.9 months vs 2.7 months, p=0.016).

All PIP patients underwent pin removal in the outpatient office, while 77% of EISN patients had surgical implant removal (p<0.001, Table 1). The mean time to implant removal was 29.8 ± 7.14 days for the PIP group, and the mean time to removal was 7.6 ± 3.37 months for the EISN group. There were no complications associated with implant removal. All fractures were followed to full healing, but the timing of implant removal resulted in a significantly shorter duration of follow-up in the PIP group (2.6 vs 7.7 months, p<0.001).

During cost analysis, institutional charges for a single implant were 10.5 times higher for ESIN than those for PIP ($598 vs $52). There were no charges for outpatient pin removal in the postoperative global period, while the mean charge for elective ESIN removal in the OR was $10,836 ± $3,891 per patient (range: $4,903-$35,121), totaling $942,756 overall cost for the entire cohort.

Thirty-eight percent (n=10) of the PIP group responded to the phone survey (Table 3) and rated pain at 3.4/10 (range: 1-8) during outpatient pin removal, with 70% rating pain as ≤3/10. One hundred percent of patients were satisfied with their overall care, and 100% of patients stated they would prefer outpatient removal to a return trip to the OR for elective operative removal (Table 3). Nine out of 10 patients reported they were “very satisfied” with their fracture treatment, while one patient, who required revision surgery for an ulnar hypertrophic nonunion, was unsatisfied with fracture management. 

**Table 3:** Modified Short Assessment of Patient Satisfaction measure (Hawthorne) for percutaneous intramedullary pinning patients/families ^a^Went on to get a revision surgery.

Question	Patient response	N=10; n (%)	Mean ± SD (range)
On a scale of 0-10, please rate the pain during implant removal in clinic:	0 = No pain	0 (0%)	3.4 ± 2.46 (1-8)
	1	2 (20%)	
	2	3 (30%)	
	3	2 (20%)	
	5	1 (10%)	
	7	1 (10%)	
	8	1 (10%)	
	9	0 (0%)	
	10 = Worst pain ever experienced	0 (0%)	
If you (or your child) had this fracture again, would you choose to have it removed in clinic again, or would you prefer to schedule surgery to have it removed with sedation?	Clinic	10 (100%)	NA
	Surgery	0 (0%)	
How satisfied are you with the effect of your fracture treatment?	0 = Very satisfied	9 (90%)	0.3 ± 0.95 (0-3)
	1 = Satisfied	0	
	2 = Neither satisfied nor dissatisfied	0	
	3 = Dissatisfied	1 (10%)^a^	
	4 = Very dissatisfied	0	
Are you satisfied with the care you received at our hospital?	0 = Very satisfied	10 (100%)	0 ± 0
	1 = Satisfied	0	
	2 = Neither satisfied nor dissatisfied	0	
	3 = Dissatisfied	0	
	4 = Very dissatisfied	0	

## Discussion

This study demonstrates that PIP is safe and effective for the treatment of radial and/or ulnar diaphyseal fractures in young patients. During the evaluation of the overall cohort and in a subanalysis of skeletally immature patients ≤10 years old, both percutaneous pinning and the more traditional buried intramedullary nail techniques have similar overall clinical and radiographic outcomes, complication profiles, and unplanned reoperations. The major benefits of a percutaneous technique are improved surgical efficiency, as we found a 40-minute shorter operative time (a finding true for both single- and bone-bone fixation), and substantial cost savings from the implant type and avoidance of planned surgical implant removal. 

Parsch published an article in which 40 patients were treated with intramedullary Kirschner wire (K-wire) fixation, all of whom had either excellent (38/40) or good outcomes [3]. Subsequent authors have demonstrated the efficacy of intramedullary percutaneous pins for the treatment of radial and/or ulnar diaphyseal fractures, with equivalent clinical and functional outcomes as ESIN and few cases of non-unions or delayed unions [4-10,14]. Most recently, Lightdale-Miric et al. compared percutaneous pins to buried intramedullary devices for treatment of Monteggia fractures, and found similar results as our study, including equivalent clinical outcomes and elimination of the need for a second surgery to remove hardware, while noting that open Monteggia fractures or patterns with a known risk of delayed union may benefit from buried instead of exposed intramedullary fixation for earlier mobilization [9]. Our study similarly found equivalent clinical outcomes when comparing percutaneous versus ESIN techniques, but ours is the first study to also add a cost analysis demonstrating the substantial cost savings of a percutaneous technique and patient-reported outcomes with avoidance of the price and anesthetic risks of staged implant removal.

Many surgeons feel that these potential risks of a retained implant outweigh the risks of implant removal, and therefore recommend routine elective implant removal. Gorter et al. performed routine planned removal of 309 ESINs at 119 mean days postoperatively, and found a 7% complication rate, thus concluding that implant removal is a safe procedure [15]. Lieber et al. found a 3.1% complication rate following removal of titanium elastic nails, and also concluded that it is a safe procedure, noting that intraoperative complications can be minimized with nail removal before signs of overgrowth [16]. Although surgical removal of an implanted flexible intramedullary nail is a relatively minor and safe procedure, it increases anesthetic exposure and potential risks to the patient, drives up cost to the family and payor, and increases the burden to the entire medical system. This was seen in our study as 77% of the 114 patients with a buried intramedullary nail required implant removal, which carried a $10,836 mean surgical charge per patient and $942,756 cost overall. In the current medical landscape of staffing shortages and increased scrutiny of medical expenses, the avoidance of unnecessary secondary surgeries may prove cost-efficient.

Leaving K-wires/Steinmann pins exposed outside the skin facilitates early removal, but concerns have been cited about potential risks [5,6,9,10,17]. Percutaneous pinning is widely used for other pediatric orthopedic injuries, such as supracondylar, lateral condyle, and distal radius fractures, with minimal risk of pin-site infection if removed by approximately four weeks [18,19]. While diaphyseal fractures may require a longer time to union, our study is consistent with other fracture patterns, as percutaneous pinning demonstrated no significant difference in infections, refractures, overall complications, time to union, or functional outcomes compared to buried implants [9,12,20]. However, in our study, percutaneous pins were selected in a younger population than ESINs, with a mean age of 6.2 years. In this young population, fracture healing is more rapid than in an older child or adolescent, so children ages 10 years and older are at a significantly higher risk of refracture [21-23]. This suggests that our finding of no refractures in the young PIP cohort should not be generalizable to older, adolescent patients. It is also notable that PIP patients were casted for an additional 2-3 weeks after pin removal to help mitigate the risk of refracture.

In addition to the potential differences in healing rate affecting implant selection, there was likely selection bias in favor of PIP for younger patients and ESIN for older patients due to the length of available implants at our institution. Only patients with a forearm length suitable for pin placement (9 in/23 cm) were included in the PIP group. When limiting the results in our study to patients ≤10 years old to capture skeletally immature patients with a potentially suitable arm length for pinning, we found similar outcomes and trends as with the entire cohort. Though Monteggia fractures may differ in fracture morphology and treatment, Monteggia fractures were included, and a subanalysis comparing Monteggia versus non-Monteggia fractures between groups showed no differences in age, surgery duration, or length of follow-up.

This study was also limited by the retrospective nature of the review. Although a subanalysis was performed for patients ≤10 years, the difference in mean age between groups could potentially introduce bias, given age-related differences in bone healing and implant selection. An additional limitation includes follow-up length, with 2.6 months on average for the PIP group. Postoperative survey results can be limited by recall bias as well as the number of patient/family responses to the survey. Additionally, patient charge data from our institution may not be generalizable across other institutions, and we were limited by a relatively small cohort of patients in the percutaneous pinning group. 

## Conclusions

In conclusion, this study demonstrates that PIP is a safe, effective, and cost-efficient technique for managing radial and ulnar diaphyseal fractures in young children with a mean age of 6.2 years, with the odds increasing by 60% for use of a buried ESIN for each year of life. Upon comparing the overall cohort and in the subanalysis of patients ≤10 years old, we found no difference between ESIN and PIP in fracture healing, complications, or unplanned reoperations. Patients who underwent percutaneous pin fixation had a 40-minute shorter duration of surgery and avoided staged surgical implant removal, which cost $10,836 per patient and $942,756 for the entire cohort. While ESIN is a well-established method of treatment with excellent outcomes, our study supports the use of percutaneous pinning as a cost-efficient alternative for diaphyseal forearm fractures in young pediatric patients with a mean age of six years.
